# Explorations on Growth of Blue-Green-Yellow-Red InGaN Quantum Dots by Plasma-Assisted Molecular Beam Epitaxy

**DOI:** 10.3390/nano12050800

**Published:** 2022-02-26

**Authors:** Xue Zhang, Zhiwei Xing, Wenxian Yang, Haibing Qiu, Ying Gu, Yuta Suzuki, Sakuya Kaneko, Yuki Matsuda, Shinji Izumi, Yuichi Nakamura, Yong Cai, Lifeng Bian, Shulong Lu, Atsushi Tackeuchi

**Affiliations:** 1 School of Nano-Tech and Nano-Bionics, University of Science and Technology of China, Hefei 230026, China; xzhang2019@sinano.ac.cn (X.Z.); zwxing2017@sinano.ac.cn (Z.X.); hbqiu2018@sinano.ac.cn (H.Q.); ygu2021@sinano.ac.cn (Y.G.); 2 Key Lab of Nanodevices and Applications, Suzhou Institute of Nano-Tech and Nano-Bionics (SINANO), Chinese Academy of Sciences (CAS), Suzhou 215123, China; ycai2008@sinano.ac.cn; 3 Department of Applied Physics, Waseda University, Tokyo 169-8555, Japan; yutasuzu@toki.waseda.jp (Y.S.); ar1ak3@toki.waseda.jp (S.K.); see8c3fed78rgl@akane.waseda.jp (Y.M.); i.shinji@akane.waseda.jp (S.I.); the-last-trial@asagi.waseda.jp (Y.N.); atacke@waseda.jp (A.T.); 4 Frontier Institute of Chip and System, Fudan University, Shanghai 200433, China; lfbian2006@sinano.ac.cn

**Keywords:** InGaN quantum dots, self-assembled growth, green light-emitting diode, plasma-assisted molecular beam epitaxy

## Abstract

Self-assembled growth of blue-green-yellow-red InGaN quantum dots (QDs) on GaN templates using plasma-assisted molecular beam epitaxy were investigated. We concluded that growth conditions, including small N_2_ flow and high growth temperature are beneficial to the formation of InGaN QDs and improve the crystal quality. The lower In/Ga flux ratio and lower growth temperature are favorable for the formation of QDs of long emission wavelength. Moreover, the nitrogen modulation epitaxy method can extend the wavelength of QDs from green to red. As a result, visible light emissions from 460 nm to 622 nm have been achieved. Furthermore, a 505 nm green light-emitting diode (LED) based on InGaN/GaN MQDs was prepared. The LED has a low external quantum efficiency of 0.14% and shows an efficiency droop with increasing injection current. However, electroluminescence spectra exhibited a strong wavelength stability, with a negligible shift of less than 1.0 nm as injection current density increased from 8 A/cm^2^ to 160 A/cm^2^, owing to the screening of polarization-related electric field in QDs.

## 1. Introduction

In recent years, red-green-blue (RGB) micro light-emitting diode (micro-LED) has been considered a promising display technology with many excellent features such as high luminescent efficiency, quick response, and long lifespan [1,2]. However, there exist two main restricting factors for the development of full-color micro-LED displays. One is the difficulty in the transfer of massive micro-LED chips, and the other one is the fabrication of efficient red micro-LED chips. Currently, commercial red LED chips are made of phosphide-based AlGaInP material. However, red AlGaInP-based LED is not the best option for the RGB micro-LED matrix, because its external quantum efficiency (EQE) would drastically reduce at micro size, and its poor mechanical properties and incompatibility with InGaN-based blue and green LEDs increase the difficulty of the pick-and-place technique [3,4,5]. In addition, InGaN-based green LED that used multi-quantum wells (MQWs) as active regions has been suffering from the “green gap” and “efficiency droop” problems; they also generally exhibit emission peak wavelength shift and color purity [6,7,8]. This is mainly due to the poor polarization field strength and crystal quality of InGaN materials with high In content, so its efficiency still needs to be improved [9,10,11]. In this regard, InGaN quantum dots (QDs) have been intensively investigated as an alternative candidate for high efficiency long wavelength light-emitters. Compared with conventional planar structures, InGaN QDs can promote indium (In) incorporation and, thereby, contribute to improving green and red light emitting. Moreover, InGaN QDs have several more excellent advantages including stronger quantum confinement effect, smaller quantum-confined Stark effect (QCSE) and lower dislocation density due to the effective strain relaxation, which are conducive to the enhancement of internal quantum efficiency (IQE) [12,13,14]. Thus, InGaN QDs could be considered as a potential material for green and red LEDs in full-color micro-LED displays.

The most widely used self-assembled growth method of InGaN QDs is based on Stranki–Krastanov (S-K) mode by metal-organic chemical vapor deposition (MOCVD) and molecular beam epitaxy (MBE), which utilizes lattice mismatch between the epitaxial layer and substrate [15,16]. Therefore, the growth window for the achievement of long-wavelength self-assembled QDs, that is, the heteroepitaxy of InGaN QDs with high In composition, is very narrow, so it is still a challenge to obtain high quality QDs. Recently, a variety of methods have been adopted to grown red and near-infrared InGaN QDs, including surface pretreatment, introducing the InGaN QW-QD coupled nanostructure, growth interruption method and photoelectrochemical etched quantum dot templates [17,18,19,20]. Despite such achievements, there remains many challenges to be conquered for self-assembled InGaN QDs, such as wavelength control and quantum efficiency to meet the requirements of actual device application. In addition, research on all-visible and long-wavelength LEDs has mainly focused on quantum wells [21,22,23]. Therefore, it is necessary to explore the growth conditions for the formation of InGaN QDs in detail and to optimize the growth method on this basis to obtain long-wavelength InGaN QDs. Herein, self-assembled InGaN QDs with varied emission wavelengths were obtained on GaN-on-sapphire substrates by plasma-assisted molecular beam epitaxy (PA-MBE). We investigated the inherent relationship between morphology and optical properties of InGaN QDs and growth parameters by atomic force microscopy (AFM), photoluminescence (PL) and time-resolved PL (TRPL) measurements, and then derived the phase diagram of InGaN QDs formation. Moreover, the nitrogen modulation epitaxy method was adopted and extended the wavelength of QDs from green to red. Finally, a green InGaN QDs LED that was prepared and exhibited a strong wavelength stability with injection current.

## 2. Experiment Details

### 2.1. Self-Assembled Growth of InGaN QDs

All InGaN QDs samples were grown on c-plane (0001) 3.5-μm-thick GaN-on-sapphire substrates by Veeco Gen 20A PA-MBE system equipped with standard Ga and In effusion cells. Active nitrogen (N_2_) was supplied by a radio-frequency plasma cell. The substates were first degreased using standard solvents, and then thermally outgassed in two steps at 200 °C for 60 min and followed by 650 °C for 30 min to eliminate surface contamination. Prior to InGaN QDs growth, two GaN buffer layers were deposited consisting of a 100-nm-thick high-temperature GaN layer grown at 740 °C and a 20-nm-thick low-temperature GaN layer grown at 650 °C. Afterwards, a series of InGaN QDs samples were grown under different growth conditions. Table 1 list the detailed growth parameters of InGaN QDs including growth temperature (Tg), growth time (tg), source beam equivalent pressure (BEP), radio-frequency power and N_2_ flux for active nitrogen (N_2_). The group-III element fluxes in BEP were regulated by controlling the temperatures of In and Ga effusion sources.

### 2.2. Measurements of Surface Morphology and Optical Properties

The surface morphology of InGaN QDs was characterized by Bruker Dimension ICON AFM. PL measurements were performed at 10 K or room temperature by tunable Ti-sapphire lasers with an excitation wavelength of 405 nm. TRPL spectra were measured using a 405 nm Ti-sapphire laser with a pulse width of 100 fs and a repetition frequency of 80 MHz, and detected with a Hamamatsu C4334-04 synchro scan streak camera with a time resolution of 15 ps.

## 3. Results and Discussion

### 3.1. Formation Mechanism of Self-Assembled InGaN QDs by PA-MBE

**Figure 1:**
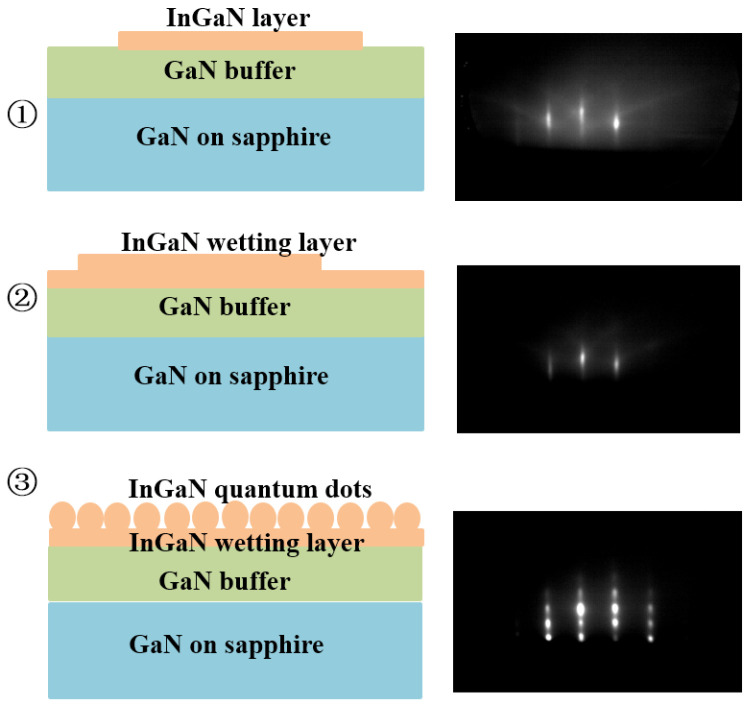
Typical RHEED images of self-assembled InGaN QDs at different growth states.

The formation of self-assembled InGaN QDs was based on S-K mode, manifesting by a spontaneous morphological transition from a two-dimensional (2D) surface to three-dimensional (3D) islands during growth after a certain thickness of InGaN wetting layer on GaN buffer. Reflection high-energy electron diffraction (RHEED) was used to real-time monitor self-forming process of InGaN QDs, as shows in Figure 1**.** The 2D-3D transition was confirmed by RHEED pattern changing from the streaky- to spotty-like. The result indicates that the formation of InGaN QDs can be achieved in conventional S-K growth method by PA-MBE.

For InGaN/GaN system, the energy of a 2D surface film and coherently strained 3D islands can respectively be described as [24],

(1) $$E_{2D}(h)=E_{2D}^{e}+E_{2D}^{s}=M\varepsilon_{InG\mathrm{aN}}^{2}h+\gamma$$

and

(2) $$E_{3D}(h)=E_{3D}^{e}+E_{3D}^{s}=(1-\alpha )M\varepsilon_{InG\mathrm{aN}}^{2}h+\gamma +\Delta \gamma$$

where *M* is the film’s biaxial modulus, *ε* is the strain, *h* is the thickness of 2D InGaN layer, *E^e^* and *E^s^* are the elastic energy for InGaN layer and the surface energy for the (0001) surface, respectively. α represents the fraction of 3D islands covering the surface. Δ*γ* is the surface energy loss to form the 3D islands. The layer will undergo a 2D-3D transition and then form InGaN QDs when *E*_3*D*_(*h*) < *E*_2*D*_(*h*) according to the lowest energy principle. In addition, islanding supposes a sufficient diffusion length that depends mainly on substrate temperature and growth rate. Therefore, it is essential to regulate the film’s free energy and facilitate the 2D-3D transition of InGaN by tuning growth parameters such as N_2_ flow and Tg.

### 3.2. Parameter Optimization of InGaN QDs

**Figure 2:**
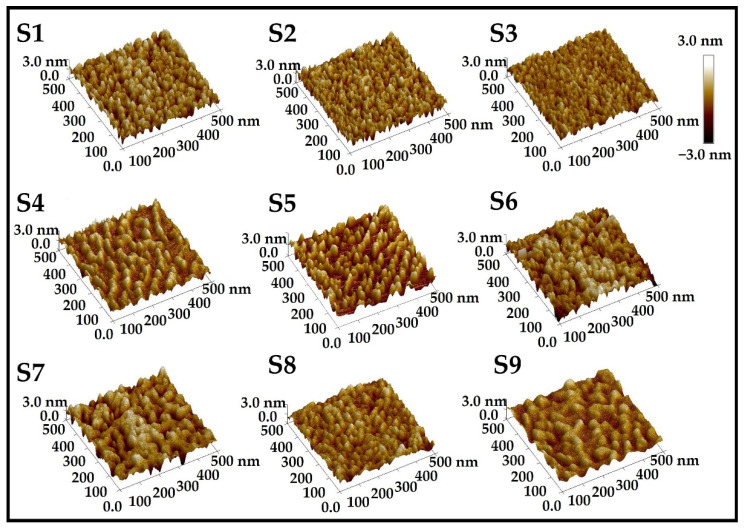
AFM 3D images of InGaN QDs grown under different conditions.

The surface morphology of InGaN QDs samples was characterized using an AFM, as shown in Figure 2. The AFM images show uniformly distributed QDs with a high surface density of over 1.0 × 10^10^ cm^−2^ for all samples, which indicates that InGaN QDs are obtained by self-assembly method. In order to explore growth of InGaN QDs, the effects of growth conditions on micromorphology and optical properties of InGaN QDs are investigated as follows.

**Figure 3:**
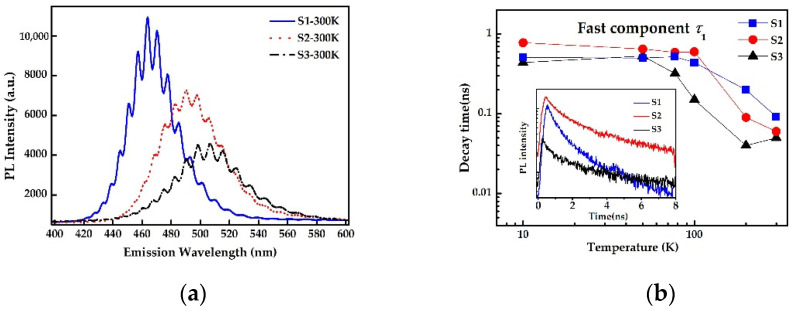
(**a**) The PL spectra of S1, S2 and S3 grown with varied In/Ga flux ratio at room temperature; (**b**) the evolution of fast carrier lifetimes for sample S1, S2 and S3 with temperature. The inset displays PL decay curves at 10 K under excited power of 3 mW.

As presented in Table 1, samples S1, S2 and S3 were grown with different In/Ga flux ratio under the premise of constant total group-III metal source, and other growth conditions remain unchanged. Room temperature PL was performed to measure optical properties of InGaN QDs grown under different growth conditions. Figure 3a shows room-temperature PL spectra of S1-S3 samples, and the interference fringes are induced by the Fabry–Perot effect [25]. It is clear that sample S1 has the best optical performance with the strongest PL intensity and the narrowest peak width, which indicates that relatively higher In/Ga ratio can improve the crystal quality of InGaN QDs. In addition, the peak wavelengths of three samples are 463 nm, 496 nm and 507 nm, respectively. That is, the PL peak wavelength of InGaN QDs appears a red-shift with decreasing the In/Ga ratio, which is opposite to that of InGaN films. The abnormal behavior of wavelength variation can be explained as the incorporation rate of Ga into InGaN crystal. In general, InGaN material should be grown at a temperature of lower than 650 °C because of high equilibrium vapor pressure of indium atoms [26]. Such a temperature is enough for In atoms during deposition, but it makes Ga atoms difficult to fully diffuse on the growth front and even incorporate into InGaN crystal under N-rich conditions. With the increase of In/Ga ratio at a given temperature, more In atoms can not only prolong diffusion length and then promote QDs formation, but also enhance the actual incorporation rate of Ga, implying shorter wavelength emission. Therefore, In/Ga flux ratio should be properly reduced to achieve longer wavelength emission, but a reasonable value is needed to ensure the quality of InGaN QDs.

Figure 3b illustrates TRPL spectra of three samples at 10 K with an excitation power of 3 mW and the variations of PL decay times with temperature. All decay curves are well fitted with two-component exponential function [27],

(3) $$I(t)=A_{1}\exp(-t/\tau_{1})+A_{2}\exp(-t/\tau_{2})$$

where *I*(*t*) is the PL intensity as a function of time, *A*_1_ and *A*_2_ are weighting coefficients, and *τ*_1_ and *τ*_2_ represent the carrier lifetimes in the fast and slow component, respectively. It is inferred that there exist two-type QD-related localization states because of size and composition fluctuations in self-assembled InGaN QDs. *τ*_1_ and *τ*_2_ correspond to the slow component in shallow localization and the fast component in deep localization, respectively. By data fitting, it was found that the value of *A*_2_ was greater or even far greater than *A*_1_ for all InGaN QDs samples in this work, which implied that the fast component dominated the decay process of photoinduced carriers. In addition, carriers’ lifetime as the key performance parameter of semiconductor materials was measured by TRPL, and the spectra were depicted in Figure 3b. It was found that carrier lifetimes for both τ_1_ and τ_2_ of three InGaN QDs samples decreased with increasing temperature, which was mainly attributed to the thermal activation of more nonradiative recombination centers. By comparison, it was found that sample S1 has the lowest sensitivity to temperature, which means the lowest nonradiative recombination. Therefore, it was further evidence for improving the crystal quality of InGaN QDs by increasing In/Ga flux ratio.

In order to explore the effect of V/III ratio on the formation of InGaN QDs, S1, S4 and S5 were grown under different N_2_ flows, as listed in the Table 1. According to AFM 3D images shown in Figure 2, we can observe that the surface morphologies of samples S4 and S5 are obviously more independent than that of sample S1, which means that high N_2_ flow is unfavorable to the formation of 3D islands. From Equations (1) and (2), it is clear that low Δγ is conducive to a 2D-3D transition. Considering that Δγ is in inverse proportional to vacuum pressure, low N_2_ flux should be applied to improve the morphology of InGaN QDs.

**Figure 4:**
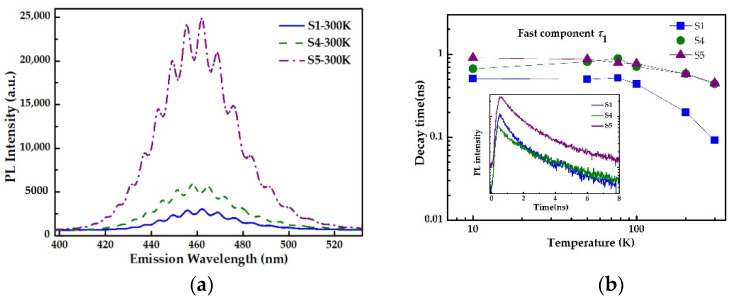
(**a**)The PL spectra of S1, S2 and S3 grown with varied N_2_ flow at room temperature; (**b**) the evolution of fast carrier lifetimes for sample S1, S4 and S5 with temperature. The inset displays PL decay curves at 10 K under excited power of 3 mW.

Room-temperature PL spectra of the three QDs samples are represented in Figure 4a. As expected, the PL intensity of InGaN QDs is markedly enhanced by decreasing N_2_ flux, while the emission wavelength shows no change. In addition, from the TRPL spectra and the variation of fitted decay times with temperature depicted in Figure 4b, it is found that lower N_2_ flux can prolong the decay time of InGaN QDs, and simultaneously reduce its sensitivity to temperature. This is mainly due to the more independent morphology of the self-assembled quantum dots formed at lower N_2_ flux, which will lead to stronger localization of carriers, and the uniformity of the quantum dots will reduce the defect density. The results demonstrate that lower N_2_ flow would improve the luminescence performance of InGaN QDs. The lifetime of samples S5 for fast decay is achieved as 450 ps at 300 K. The radiative lifetime is 910 ps on the assumption that the non-radiative recombination is fully suppressed at 10 K, which is very close to the relatively good results of InGaN QDs or QW LEDs on c-plane substrates [12,22,28]. The results indicate that QDs can reduce the built-in electric field induced by polarizations and, thus, can result in shorter lifetime. Within the above analysis, it is concluded that the N_2_ flux should be minimized on the premise of N-rich condition for the formation of self-assembled InGaN QDs.

**Figure 5:**
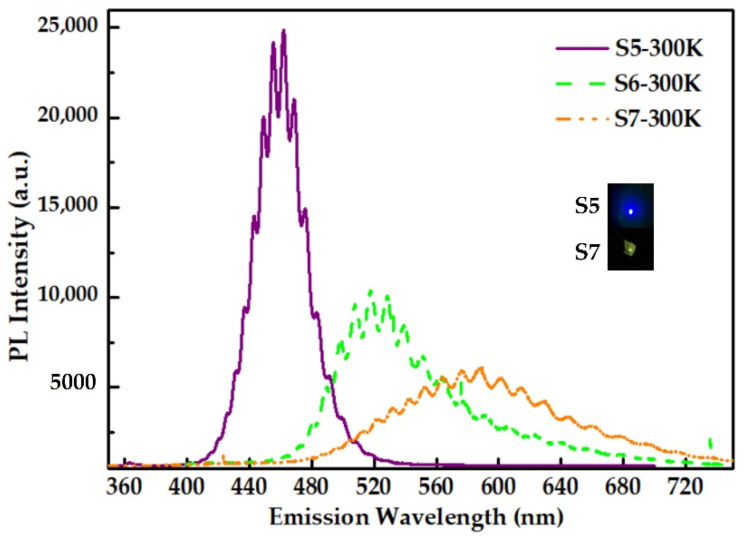
The PL spectra of S5, S6 and S7 grown with varied growth temperatures at room temperature and the insets are photographs of the resulting luminescence.

For the self-assembled growth of InGaN QDs, growth temperature is undoubtedly another key parameter. High temperature can increase diffusion length of In and Ga ad-atom on the growth front and then promote QDs formation [29]. However, high temperature is not conducive to the incorporation of In and, hence, long-wavelength emission of InGaN material, because the evaporation of metal atoms would be enhanced significantly with increasing Tg. A group of samples S6, S7 and S8 have been grown at different Tg, and the specific growth parameters have been listed in the Table 1. The peak wavelengths of the three samples shown in Figure 5 are 462 nm, 523 nm, and 581 nm, respectively, and the insets are photographs of the resulting luminescence by S5 and S7. Apparently, the emission wavelengths of InGaN QDs have shown red shift with decreasing growth temperature. As previously mentioned, emission wavelength of InGaN QDs is very sensitive to the growth temperature. In addition, the truncated pyramidal-shaped feature of self-assembled QD structure with excellent independence is exhibited in blue InGaN QDs, which is better than longer-wavelength samples, according to 3D AFM images in Figure 2. It was confirmed that high temperature was beneficial to improving the micro-topography of InGaN QDs. In conclusion, Tg should be optimized to achieve long-wavelength emission without high evaporation rates, while ensuring sufficient diffusion length to improve crystal quality.

### 3.3. Phase Diagram of InGaN QDs Grown by PA-MBE

**Figure 6:**
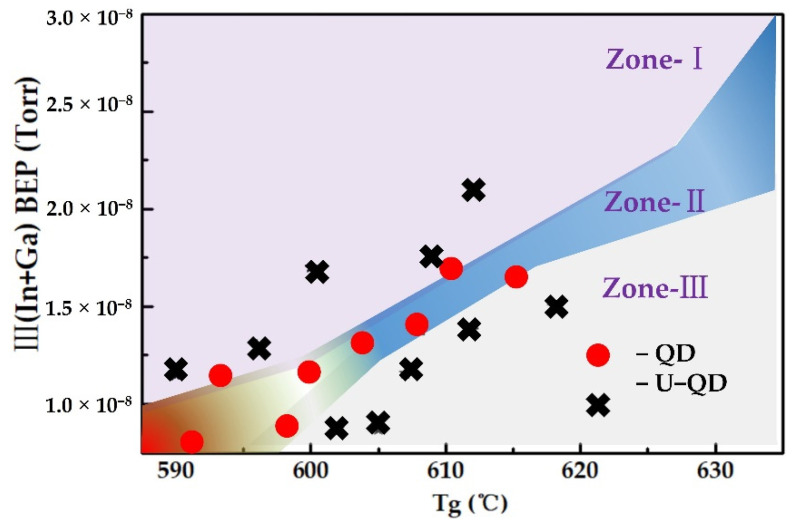
Phase diagram of InGaN quantum dot material growth.

More InGaN QDs samples have been grown to probe into the growth conditions for the spontaneous formation of self-assembled InGaN QDs by SK mode. Based on above experiments and analysis results, we derived the phase diagram of InGaN QDs grown by PA-MBE, as illustrated in Figure 6, when the nitrogen flow rate and plasma power were fixed at 1.0 sccm and 400 W, respectively. In the first regime of phase diagram noted as Zone-I, low Tg and high total metal BEP led to high Δ*γ* and, thus, E_3D_(h) > E_2D_(h), resulting in 2D surface growth of InGaN material. Furthermore, under high Ts and low metal BEP in Zone-III regime, it was difficult to from 3D islands because of high evaporation rates. Therefore, the growth window of QD islands was very narrow. More specifically, the metal BEP was less than 3 × 10^−8^ torr, while growth temperature was lower than 630 °C.

### 3.4. Nitrogen Modulation Epitaxy Method Based on SK Mode

**Figure 7:**
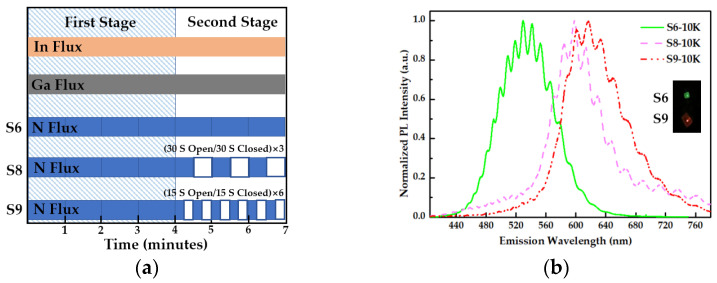
(**a**) Schematic diagram of nitrogen modulation epitaxy method; (**b**) The normalized PL emission spectrum of S6, S8, S9 at 10 K and the insets are photographs of the resulting luminescence.

From the growth phase diagram of InGaN quantum dots grown by MBE, it can be seen that the window conditions for the growth of long-wavelength InGaN QDs were relatively narrow, so the growth method needed to be optimized. The nitrogen modulation epitaxy method is based on the SK mode, that is, the growth process is divided into two stages. The schematic diagram of the nitrogen modulation epitaxy method is shown in Figure 7a. In the first stage, it was ensured that a wetting layer was formed to realize the self-assembly growth of QDs under the condition of slight nitrogen enrichment, as shown in Figure 1. ①. This process also includes the RHEED diffraction pattern from the streaky- to spotty-like, that is, abrupt change from ② to ③ in Figure 1. According to the monitoring of the samples’ growth process, these stages usually lasted for about 4 min. In the second stage, the nitrogen source is periodically turned on and off during the growth process, while the metal sources of In and Ga are always maintained. In the second stage, the nitrogen source must be periodically turned on and off. Turning on the nitrogen source ensured nitrogen-rich conditions for growing quantum dots in SK mode, while, when the nitrogen source was turned off, the reduction in the number of nitrogen atoms promoted the formation of 3D islands. At the same time, the metal atoms left on the growth surface had enough time to diffuse laterally, promoting the increase of In composition to red-shift the wavelength.

The growth parameters of samples S6, S8 and S9 are listed in the Table 1 and the growth process is shown in Figure 7a; the three samples were kept the same in the first stage. The difference is that, in the second stage, the N source of sample S6 was always on, while the on and off times of sample S8 were 30 s for 3 cycles, respectively, while the on and off times of sample S9 were 15 s for 6 cycles. Figure 5 is the normalized PL emission spectrum of S6, S8, S9 at 10 K, and the insets were photographs of the resulting luminescence by S6 and S9; in addition, the peak wavelengths were 523 nm, 598 nm, and 622 nm, respectively. The more cycles of the N source turned on and off, the better the wavelength redshift, so that the wavelength of InGaN QDs was shifted from green to red. Therefore, the nitrogen modulation epitaxy method was an effective solution for obtaining InGaN QDs emitting light in the red regions.

### 3.5. Fabrication of InGaN QDs Green LED

**Figure 8:**
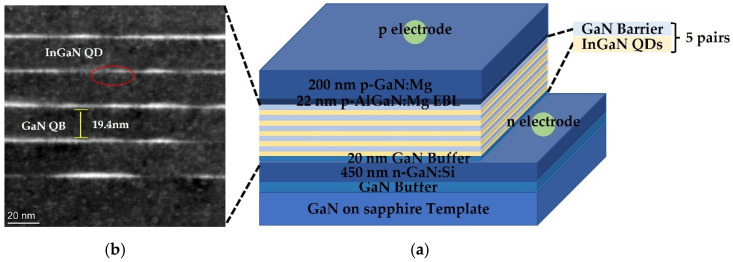
(**a**) Schematic diagram of the QD LED structure and (**b**) Cross-sectional TEM image of InGaN/GaN MQDs region in LED.

The schematic structure of InGaN QDs LED is shown in Figure 8a. A total of 450 nm Si-doped and 200 nm Mg-doped GaN were used as n-type and p-type contact layers, respectively. InGaN/GaN multilayers quantum dots (MQDs) structure with five pairs was employed as the active region of InGaN-based LED. GaN quantum barrier (QB) layers were grown at low temperature of 650 °C to protect InGaN QDs. A 22 nm-thick AlGaN layer was applied as electron blocking layer (EBL) to reduce electron leakage. Figure 8b shows the cross-sectional TEM image of InGaN/GaN MQDs structure in QDs LED. We could clearly observe that distinct InGaN QDs were scattered in GaN barrier layers. The height of the QD marked in red circle was about 2.6 nm. In addition, the thickness of the GaN QB layer was about 19.4 nm, and the InGaN/GaN interface was very abrupt. Afterwards, an InGaN-based LED was fabricated by using conventional LED process. However, it should be noted that current spreading layer was not introduced in device fabrication. Finally, the epilayers were divided into devices with 250 μm × 250 μm.

**Figure 9:**
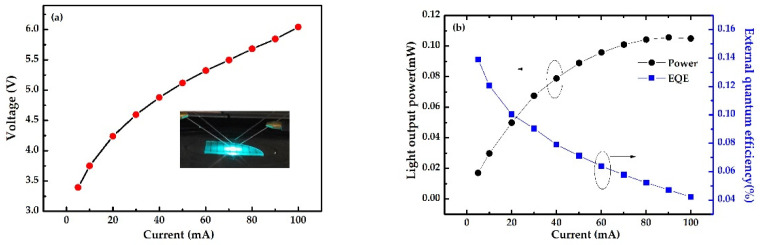

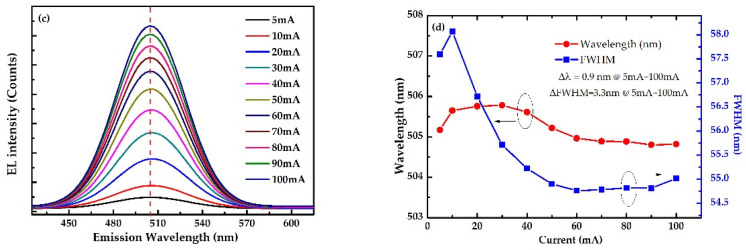
(**a**) Forward voltage versus injection current; (**b**) LOP and EQE versus injection current; (**c**) EL spectra of QD LED under injection current from 5 mA to 100 mA; (**d**) the variation of EL peak wavelength and FWHM with injection current.

Figure 9a,b show the variations of forward voltage, light output power (LOP) and external quantum efficiency (EQE), respectively, with injection current from 5 mA to 100 mA, i.e., corresponding current density from 8 A/cm^2^ to 160 A/cm^2^. The LOP increased firstly and then tended to be stabilized gradually with the increase of injection current. The QD LED presented a significant efficiency droop; the efficiency of QD LED was as low as 0.14% at injection current of 5 mA. The efficiency droop at high current owed to the large non-radiative recombination rate. We inferred that poor performance may be attributed to following possible reasons. One is that thick GaN QB layers grown at low temperature produced high-density defects, which resulted in nonradiative recombination, as well as carrier delocalization [30,31]. The other is that severe current crowding (CC) occurring in p-GaN without current spreading layer enhanced Auger recombination and electron leakage [32], which are underling mechanisms of the efficiency recombination in lateral GaN-based LEDs [30,33]. Furthermore, because QDs have lower electron capture cross section that QWs, the low capture probability would reduce the efficiency of LED. In addition, electroluminescence (EL) measurements under various injection current at room temperature were performed on the LED. As depicted in Figure 9c,d, the peak wavelength located at 505 nm with a shift of less than 1.0 nm as the injection current was gradually increased from 5 mA to 100 mA. It can be compared with green semipolar InGaN QW LEDs [34]. The results showed excellent wavelength stability of LED, which was mainly attributed to the fully screened polarization-related electric field and, hence, to suppressed QCSE in InGaN QDs [12,35]. The epitaxial structure and fabrication process would be further optimized in our future work to overcome efficiency droop and to improve the performance of InGaN-based green LEDs.

## 4. Conclusions

In summary, we explored S-K growth of self-assembled InGaN QDs on GaN-on-sapphire templates using PA-MBE by regulating In/Ga flux ratio, N_2_ flow and growth temperature. The effects of growth parameters on the spontaneous formation of InGaN QDs were investigated by AFM and PL measurements; consequently, the phase diagram of MBE-grown InGaN QDs was described. Small N_2_ flow is instrumental in improving morphology and luminescent properties of InGaN QDs, whereas low growth temperature and In/Ga flux ratio are applicable for long-wavelength emission. As a result, InGaN QDs with light emission from 460 nm to 622 nm were achieved by tuning growth conditions and with nitrogen modulation epitaxy method. In addition, a 505 nm green LED based on InGaN/GaN MQDs was prepared. The LED had a low EQE of less than 0.14% and showed a strong efficiency droop with increasing injection current. However, the EL emission wavelength exhibited a minuscule shift of less than 1nm as injection current was increased from 5 mA to 100 mA, which indicated that the built-in filed was screened well in InGaN QDs.

## Figures and Tables

**Table 1:** The growth parameters of all samples.

Sample	Tg/(°C)	N_2_ Flux/Power (Sccm/W)	Tg/(min)	In BEP/(torr)	Ga BEP/(torr)	λ@RT/(nm)
S1	610	1.5/430	7	1.2 × 10^−8^	4 × 10^−9^	463
S2	610	1.5/430	7	1 × 10^−8^	6 × 10^−9^	496
S3	610	1.5/430	7	9 × 10^−9^	7 × 10^−9^	507
S4	610	1.0/400	7	1.2 × 10^−8^	4 × 10^−9^	460
S5	610	0.6/350	7	1.2 × 10^−8^	4 × 10^−9^	462
S6	605	1.5/430	7	9 × 10^−9^	3 × 10^−9^	523
S7	595	1.5/430	7	7.5 × 10^−9^	2.5 × 10^−9^	581
S8	605	1.5/430	4 + (30 s/30 s) ∗ 3	9 × 10^−9^	3 × 10^−9^	598
S9	605	1.5/430	4 + (15 s/15 s) ∗ 6	9 × 10^−9^	3 × 10^−9^	622

## Data Availability

The data presented in this study are available within the manuscript.
